# Temporal Trends in Complex Percutaneous Coronary Interventions

**DOI:** 10.3389/fcvm.2022.913588

**Published:** 2022-06-24

**Authors:** Mark Kheifets, Shelly Abigail Vons, Tamir Bental, Hana Vaknin-Assa, Gabriel Greenberg, Abed Samara, Pablo Codner, Guy Wittberg, Yeela Talmor Barkan, Leor Perl, Ran Kornowski, Amos Levi

**Affiliations:** ^1^Department of Cardiology, Rabin Medical Center, Petach Tikva, Israel; ^2^Affiliated to the Faculty of Medicine, Tel Aviv University, Tel Aviv, Israel

**Keywords:** trends, complexity, PCI—percutaneous coronary intervention, bifurcation, CTO (chronic total occlusion), left main, SVG = saphenous vein graft

## Abstract

**Background:**

Accumulated experience combined with technological advancements in percutaneous coronary interventions (PCI) over the past four decades, has led to a gradual increase in PCI utilization and complexity. We aimed to investigate the temporal trends in PCI complexity and the outcomes of complex PCI (C-PCI) in our institution.

**Methods:**

We analyzed 20,301 consecutive PCI procedures performed over a 12-year period. C-PCI was defined as a procedure involving at least one of the following: Chronic total occlusion (CTO), left main (LM), bifurcation or saphenous vein graft (SVG) PCI. Four periods of 3-year time intervals were defined (2008–10, 2011–2013, 2014–2016, 2017–2019), and temporal trends in the rate and outcomes of C-PCI within these intervals were studied. Endpoints included mortality and major adverse cardiac events [MACE: death, acute myocardial infarction (MI), and target vessel revascularization (TVR)] at 1 year.

**Results:**

A total of 5,647 (27.8%) C-PCI procedures were performed. The rate of C-PCI has risen significantly since 2,017 (31.2%, *p* < 0.01), driven mainly by bifurcation and LM interventions (*p* < 0.01). At 1-year, rates of death, acute MI, TVR and MACE, were all significantly higher in the C-PCI group (8.8 vs. 5.1%, 5.6 vs. 4.5%, 5.5 vs. 4.0%, 17.2 vs. 12.2%, *p* < 0.001 for all, respectively), as compared to the non-complex group. C-PCI preformed in the latter half of the study period (2014–2019) were associated with improved 1-year TVR (4.4% and 4.8% vs. 6.7% and 7.1%, *p* = 0.01, respectively) and MACE (13.8% and 13.5% vs. 17.3% and 18.2%, *p* = 0.001, respectively) rates compared to the earlier period (2007–2013). Death rate had not significantly declined with time.

**Conclusion:**

In the current cohort, we have detected a temporal increase in PCI complexity coupled with improved 1-year clinical outcomes in C-PCI.

## Introduction

Complex percutaneous coronary intervention (C-PCI) is commonly defined as an elective or urgent PCI with any of the following characteristics: ≥ 3 drug eluting stents (DES) implanted, bifurcation PCI with 2 stents, left main (LM) coronary artery PCI, saphenous vein graft (SVG) PCI, total stent length > 60 mm, or chronic total occlusion (CTO) as target lesion (1). Patients who undergo C-PCI with DES in the setting of both stable coronary artery disease (CAD) and acute coronary syndrome (ACS), are at a substantially higher ischemic risk, in a graded fashion, with increased procedural complexity (1, 2). As clinical and angiographic characteristics of patients undergoing PCI with DES have evolved substantially over the last 20 years, current trends indicate that approximately 30% of all PCI procedures may be considered complex according to lesion or anatomic factors (3). Owing to technical and methodological advancements, patients who were previously treated medically or surgically, are now often offered PCI with an emphasis on unprotected left main disease (ULMD) (4–6) and CTO (7). In order to successfully predict and identify patients who are prone to increased residual ischemic risk, these intricate procedures require interventional cardiologists to use both clinical (8) and angiographic (9) risk scores, and implement treatment accordingly. The aim of the current study was to evaluate the trends of complex PCI procedures throughout the last decade, with their subsequent outcomes. Specifically, we sought to compare trends in a large prospective registry of patients treated at an academic medical center institution which encompasses 2 hospitals.

## Materials and Methods

### Patients and Setting

All consecutive patients who underwent PCI at the Rabin Medical Center (RMC), Petach Tikva, Israel (“Hasharon” and “Beilinson” campuses) between January 2008 and December 2019 were included in the current analysis. Data regarding clinical diagnoses were collected from the institutional electronic medical record system, in keeping with the ICD-9 system. Laboratory data were retrieved from the RMC central laboratory database. Demographic data, including death dates, were obtained from the institutional demographic information system, which is linked to the state of Israel Ministry of Interior data, and was thereafter verified with the Israel Central Bureau of Statistics. Patients’ follow-up was performed using a detailed registry, collected from the institutional electronic medical records system.

### Clinical and Procedural Data

All follow-up data were collected up to June 2021. Data collection was approved by the institutional ethics committee in compliance with the Declaration of Helsinki, with a waiver for the need of individual informed consent. We initially compared C-PCI and non-C-PCI patients. C-PCI was defined as a procedure involving at least one of the following: CTO, LM, bifurcation or SVG PCI. Four periods of 3-year time intervals were defined (2008–2010, 2011–2013, 2014–2016, 2017–2019), and temporal trends in the rate and outcomes of C-PCI within these intervals were studied.

### Clinical Endpoints

Endpoints included mortality and major adverse cardiac events [MACE: death, acute myocardial infarction (MI), and target vessel revascularization (TVR)] at 1 year. In accordance with the “fourth universal definition of myocardial infarction” (10), MI was defined as acute myocardial injury with clinical evidence of acute myocardial ischemia and with detection of a rise and/or fall of cTn values with at least one value above the 99th percentile URL and at least one of the following:

• Symptoms of myocardial ischemia.
• New ischemic ECG changes.
• Development of pathological Q waves.
• Imaging evidence of new loss of viable myocardium or new regional wall motion. abnormality in a pattern consistent with an ischemic etiology.
• Identification of a coronary occlusion or significant stenosis by angiography.

To evaluate the interaction between complexity and temporal trends in outcomes (i.e., death and MACE) additional multivariate cox models were constructed with time period as a continuous variable and a complexity: time period interaction term.

### Statistical Analysis

Categorical data are reported as frequency and percentages and compared using the χ^2^-test or the Fisher exact test, as appropriate. Continuous variables are presented as mean ± *SD* or median and interquartile range and compared using the 2-sample *t*-test or the 2-sample Wilcoxon rank-sum (Mann-Whitney) test. All tests were two-tailed, and *p* < 0.05 was considered significant. Analysis of between period trends were performed using the Cochran-Armitage trend test or linear regression as appropriate.

Time-to-event curves were constructed using the Kaplan–Meier method and compared using the log-rank test. Given competing risks, cumulative incidence functions were used to plot 1 year risk of TVR and acute MI. Univariate and multivariate Cox regression models were conducted to evaluate the association between complexity and outcomes, and the association between time period and outcomes within the complex PCI group. The following covariates were included in the multivariable model: age, hypertension, diabetes mellitus (DM) congestive heart failure (CHF), severe left ventricular (LV) systolic function, prior myocardial infarction (MI) or ACS, cardiogenic shock and renal failure. Covariate were selected owing to uneven distribution between study groups. All analyses were performed using R (R-studio, V.4.0.0, Vienna, Austria).

## Results

### Patients and Procedural Characteristics

Of 20,301 procedures performed over a period of 12 years (2008–2020), 5,647 (27.8%) were identified as complex. Baseline characteristics of complex vs. non-complex PCIs are shown in Table 1. There was no difference in gender distribution or smoking between the groups (*p* = NS for both). Patients in the C-PCI group were older (66.3 ± 12.6 vs. 65.6 ± 11.9, *p* < 0.001), had lower estimated glomerular filtration rate (eGFR) (79.8 ± 29.0 vs. 82.1 ± 28.6, *p* < 0.001), were more likely to have CHF (12.4 vs. 10.6%, *p* < 0.001) and severe LV systolic function (21.4 vs. 15.9%, *p* < 0.001), were more likely to present as ST-elevation myocardial infarction (STEMI) (20.9 vs. 11.2%, *p* < 0.001) or cardiogenic shock (2.5 vs. 0.8%, *p* < 0.001), were more likely to be treated using anti-coagulation therapy (10.4 vs. 8.5%, *p* < 0.001), had a higher rate of significant proximal left anterior descending (LAD) disease (24.5 vs. 18.4%, *p* < 0.001), were less likely to be catheterized *via* transradial approach (45.0 vs. 50.6%, *p* < 0.001), and were treated with a IIbIIIa inhibitors more frequently (11.9 vs. 7.5%, *p* < 0.001), as compared to the non-complex PCI group. Baseline characteristics of patients who underwent C-PCI procedures, grouped to 4 periods (2008–2010, 2011–2013, 2014–2016, 2017–2019), are shown in Table 2. There was no difference between the groups in gender distribution, smoking rate, DM rate or eGFR (*p* = NS for all). A positive temporal trend was observed in patient age (65.1 ± 12.7 vs. 66.5 ± 12.9 vs. 67.3 ± 12.4 vs. 66.3 ± 12.5, respectively, *p* = 0.01), rate of hypertension (79.3% vs. 76.4% vs. 74.8% vs. 70.2%, respectively, *p* < 0.001), significant proximal LAD disease (20.2% vs. 20.8% vs. 24.4% vs. 30.3%, respectively, *p* < 0.001), and transradial approach rate (3.4% vs. 31.0% vs. 64.7% vs. 69.7%, respectively, *p* < 0.001), while a negative temporal trend was detected in the rate of CHF (18.3% vs. 16.1% vs. 10.7% vs. 6.9%, respectively, *p* < 0.001), previous CABG (25.8% vs. 22.3% vs. 18.7% vs. 14.8%, respectively, *p* < 0.001), cardiogenic shock on presentation (3.2% vs. 3.0% vs. 2.1% vs. 2%, respectively, *p* < 0.05) and IIbIIIa inhibitor administration (26.9% vs. 15.8% vs. 6.8% vs. 5.3%, respectively, *p* < 0.001).

**TABLE 1:** Baseline characteristics, complex vs. non-complex PCI.

Variable	Non-complex 14,654 (72.2%)	complex 5,647 (27.8%)	*P-*value
Age (years)	65.6 ± 11.9	66.3 ± 12.6	<0.001
Female gender	3,194 (21.8)	1,208 (21.4)	0.56
Hypertension	11,312 (77.2)	4,218 (74.7)	<0.001
Smoking Hx	5,305 (36.2)	2,010 (35.6)	0.37
Diabetes mellitus	7,239 (49.4)	2,677 (47.4)	0.01
Dementia	278 (1.9)	136 (2.4)	0.02
CHF	1,553 (10.6)	700 (12.4)	<0.001
Prior CABG	1,597 (10.9)	1,124 (19.9)	<0.001
Anticoagulation	1,246 (8.5)	587 (10.4)	<0.001
Hemoglobin	13.3 ± 1.8	13.2 ± 1.8	0.12
eGFR	82.1 ± 28.6	79.8 ± 29.0	<0.001
Prior MI or ACS	8,133 (55.5)	3,569 (63.2)	<0.001
Proximal LAD	2,696 (18.4)	1,383 (24.5)	<0.001
Radial access	7,415 (50.6)	2,541 (45)	<0.001
Severe LV function	2,330 (15.9)	1,207 (21.4)	<0.001
IIbIIIa inhibitor administration	1,099 (7.5)	672 (11.9)	<0.001
Cardiogenic shock	117 (0.8)	141 (2.5)	<0.001

*Values are expressed as n (%) or mean ± SD.*

*PCI, percutaneous coronary intervention; CHF, congestive heart failure; CABG, coronary artery bypass graft; eGFR, estimated glomerular filtration rate; MI, myocardial infarction; ACS, acute coronary syndrome; LAD, left anterior descending; LV, left ventricle.*

**TABLE 2:** Baseline characteristics of complex PCI per period.

Variable	2008–2010 1,263 (22.4%)	2011–2013 1,232 (21.8%)	2014–2016 1,387 (24.6%)	2017–2019 1,765 (31.2%)	*P-*value
Age (years)	65.1 ± 12.7	66.5 ± 12.9	67.3 ± 12.4	66.3 ± 12.5	0.01
Female gender	251 (19.9)	278 (22.6)	301 (21.7)	379 (21.5)	0.50
Hypertension	1,002 (79.3)	941 (76.4)	1,037 (74.8)	1,239 (70.2)	<0.001
Smoking Hx	462 (36.6)	400 (32.5)	456 (32.9)	688 (39)	0.12
Diabetes mellitus	623 (49.3)	580 (47.1)	642 (46.3)	830 (47.0)	0.28
Dementia	33 (2.6)	25 (2.0)	33 (2.4)	44 (2.5)	0.89
CHF	231 (18.3)	198 (16.1)	148 (10.7)	122 (6.9)	<0.001
Prior CABG	326 (25.8)	275 (22.3)	259 (18.7)	261 (14.8)	<0.001
Anticoagulation	130 (10.3)	136 (11.0)	122 (8.8)	199 (11.3)	0.73
Hemoglobin	13.2 ± 1.8	13.2 ± 1.9	13.3 ± 1.8	13.3 ± 1.8	0.92
eGFR	82.2 ± 28.1	81.6 ± 30.5	77.6 ± 27.7	78.6 ± 29.3	0.59
Prior MI or ACS	820 (64.9)	777 (64.9)	859 (61.9)	1,114 (63.1)	0.36
Proximal LAD	255 (20.2)	256 (20.8)	338 (24.4)	535 (30.3)	<0.001
Radial access	43 (3.4)	382 (31.0)	897 (64.7)	1,220 (69.1)	<0.001
Severe LV function	259 (20.5)	269 (21.8)	275 (19.8)	408 (23.1)	0.23
IIbIIIa inhibitor administration	340 (26.9)	195 (15.8)	94 (6.8)	94 (5.3)	<0.01
Cardiogenic shock	40 (3.2)	37 (3.0)	29 (2.1)	35 (2)	0.04

*Values are expressed as n (%) or mean ± SD.*

*PCI, percutaneous coronary intervention; CHF, congestive heart failure; CABG, coronary artery bypass graft; eGFR, estimated glomerular filtration rate; MI, myocardial infarction; ACS, acute coronary syndrome; LAD, left anterior descending; LV, left ventricle.*

### Temporal Trends of Clinical Outcomes

**FIGURE 1:**
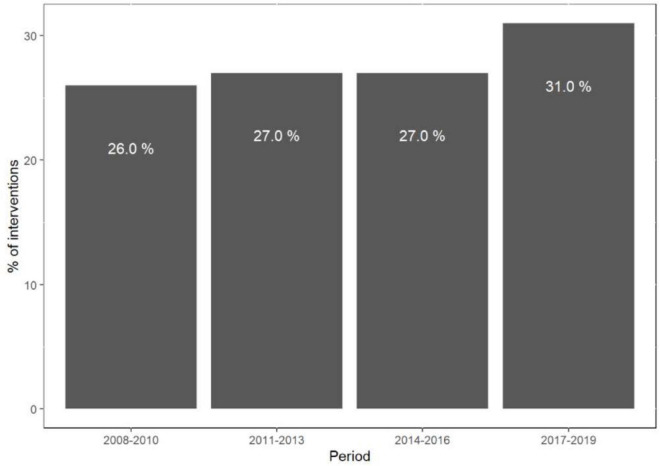
Rate of complex PCI (percent of total interventions) per time period.

**FIGURE 2:**
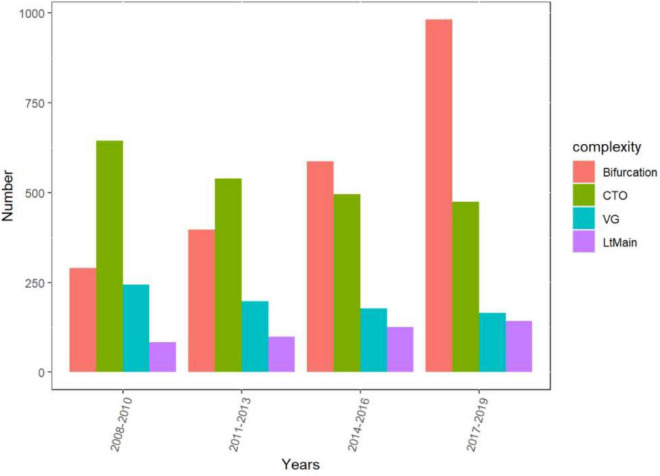
Interventions grouped by time period and complexity definition.

The rate of C-PCI procedures has risen significantly since 2017 (*p* < 0.01) (Figure 1), mainly driven by bifurcation and LM interventions (*p* < 0.01), whereas the amount of SVG and CTO procedures has steadily declined (*p* < 0.01) (Figure 2).

**FIGURE 3:**
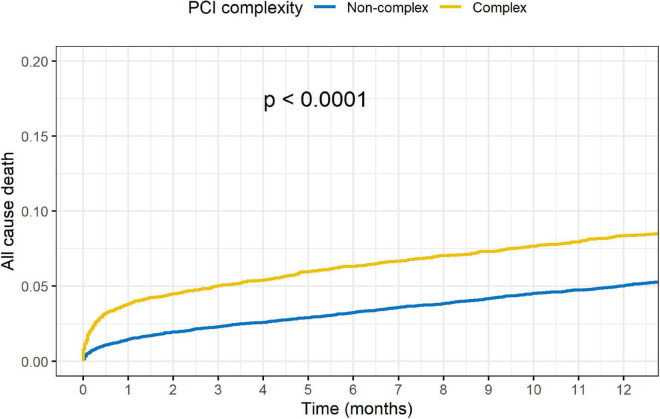
Complex vs. non-complex (death).

**FIGURE 4:**
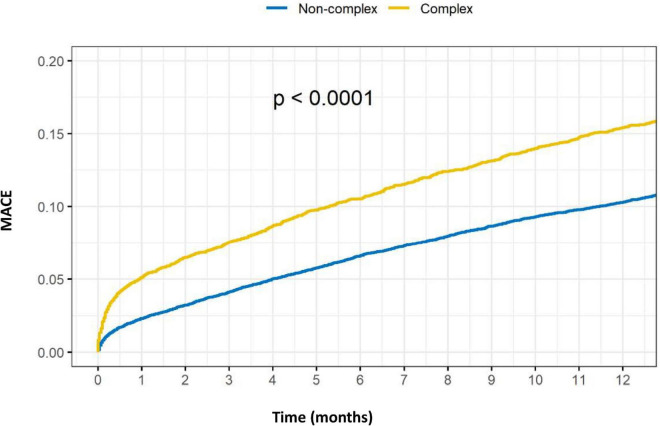
Complex vs. non-complex (MACE).

**FIGURE 5:**
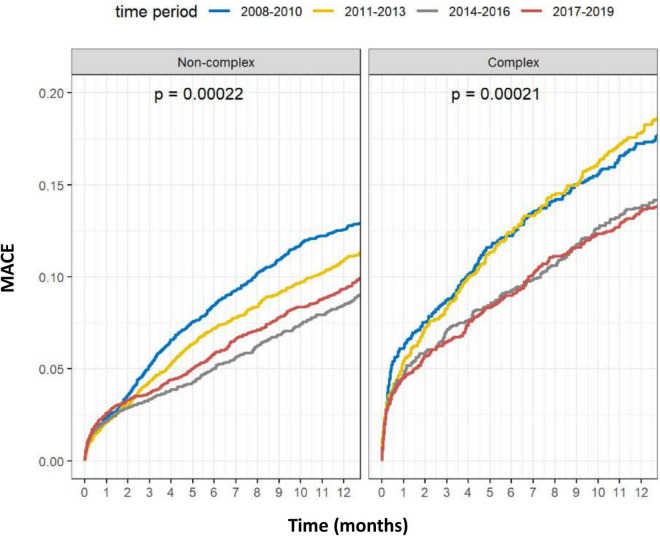
Complex vs. non-complex (per time period MACE).

**FIGURE 6:**
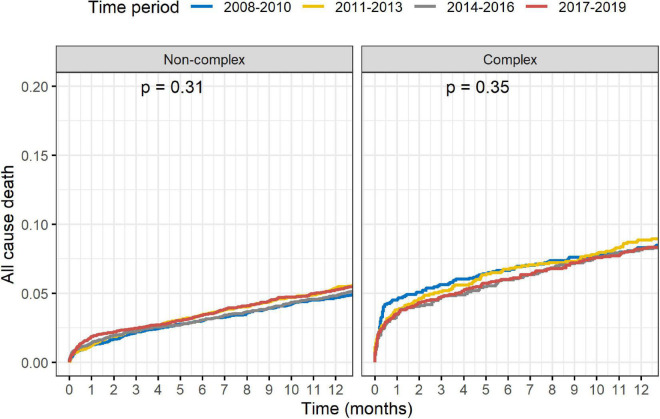
Complex vs. non-complex (per time period death).

Outcomes of complex vs. non-complex PCIs are shown in Table 3. At 1-year, rates of death (8.4 vs. 5.1%, *p* < 0.001), acute MI (5.5 vs. 4.0%, *p* < 0.001), TVR (5.6 vs. 4.5%, *p* = 0.001) and MACE (15.4 vs. 10.3%, *p* < 0.001), were all significantly higher in the C-PCI group, as compared to the non-complex group (Figures 3, 4). Notably, even though MACE was significantly lower in the later periods in both complex and non-complex groups (Figure 5), overall all-cause mortality did not change during the study period, regardless of complexity (Figure 6). Interaction between C-PCI and MACE (HR: 0.88, 95% CI 0.84–0.92, *p* < 0.001) was found to be significant. In contrast, interaction between C-PCI and death (HR: 1.02, 95% CI 0.96–1.09, *p* = 0.444) was not significant.

**TABLE 3:** Complex vs. non-complex outcomes.

Variable	Non-complex 14,654 (72.2%)	Complex 5,647 (27.8%)	*P-*value
Death	746 (5.1)	474 (8.4)	<0.001
TVR	654 (4.5)	317 (5.6)	<0.001
Acute MI	580 (4.0)	311 (5.5)	<0.001
MACE	1,515 (10.3)	872 (15.4)	<0.001

*Values are expressed as n (%) or mean ± SD.*

*PCI, percutaneous coronary intervention; TVR, Target vessel revascularization MI, myocardial infarction; MACE, major adverse cardiovascular event.*

**FIGURE 7:**
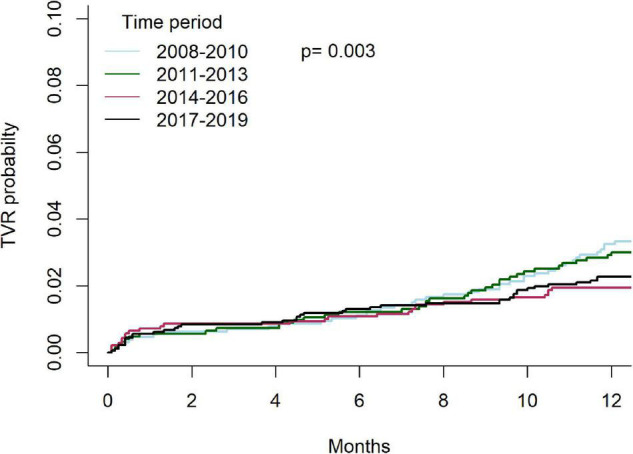
Cumulative incidence of TVR at 1 year, grouped by time period.

**FIGURE 8:**
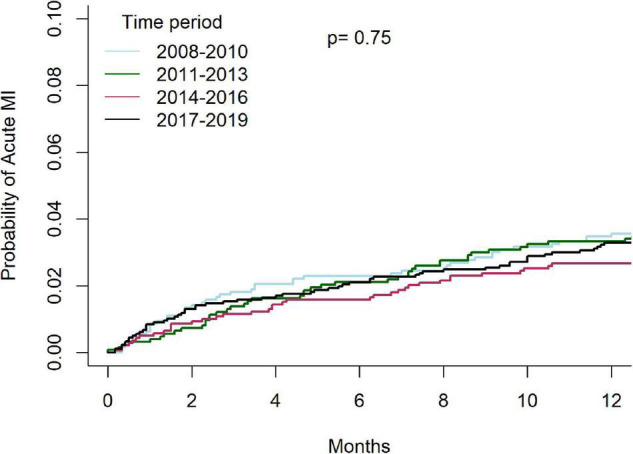
Cumulative incidence of acute-MI at 1 year, grouped by time period.

Temporal outcomes of patients who underwent C-PCIs are shown in Table 4. Rates of MACE at 1-year were significantly lower in both the 2014–2016 and 2017–2019 groups, as compared to the 2008–2010 and 2011–2013 groups (13.8% and 13.5% vs. 17.3% and 18.2%, *p* = 0.001, respectively). This was driven by lower rates of TVR at 1-year (4.4% and 4.8% vs. 6.7% and 7.1%, *p* = 0.01, respectively). There was no difference in rates of acute MI (4.6% vs. 5.9% vs. 5.2% vs. 6.2%, *p* = 0.76, respectively) or death (8.2% vs. 8.3% vs. 8.3% vs. 8.8%, *p* = 0.83, respectively) at 1-year between the groups. Cumulative incidence of TVR and acute-MI at 1 year, grouped by time periods, are shown in Figures 7, 8, respectively.

**TABLE 4:** Complex PCI—temporal outcomes.

Variable	2008–2010 1,263 (22.4%)	2011–2013 1,232 (21.8%)	2014–2016 1,387 (24.6%)	2017–2019 1,765 (31.2%)	*P-*value
Death	105 (8.3)	109 (8.8)	114 (8.2)	146 (8.3)	0.83
TVR	84 (6.7)	87 (7.1)	61 (4.4)	85 (4.8)	0.01
Acute MI	66 (5.2)	76 (6.2)	64 (4.6)	105 (5.9)	0.76
MACE	218 (17.3)	224 (18.2)	192 (13.8)	238 (13.5)	0.001

*Values are expressed as n (%) or mean ± SD.*

*PCI, percutaneous coronary intervention; TVR, Target vessel revascularization MI, myocardial infarction; MACE, major adverse cardiovascular event.*

The results of the COX regression analyses are shown in Tables 5, 6. After adjustment for possible confounders, C-PCI was an independent risk factor for both death (HR: 1.34, 95%CI 1.19–1.51, *P* < 0.001) and MACE (HR- 1.30, 95%CI 1.19–1.42, *P* < 0.001). As to the effect of time periods on outcomes of C-PCI: only the most recent time period (2017–2019) emerged as an independent prognostic variable of lower MACE rate (compared to 2008–2011, HR: 0.79, 95% CI 0.66–0.96, *p* = 0.015). Notably, a trend toward lower 1-year MACE was observed throughout the study period (HR: 0.91, 95%CI 0.86–0.97, *p* for trend = 0.02).

**TABLE 5:** Cox regressions models—complex vs. non-complex PCI.

Outcome	HR (95% CI)	*P-*value
Death univariate	1.69 (1.50–1.89)	<0.001
Death multivariate*	1.34 (1.19–1.51)	<0.001
MACE univariate	1.55 (1.42–1.68)	<0.001
MACE multivariate*	1.30 (1.19–1.42)	<0.001

*Adjusted to—age, hypertension, diabetes mellitus, congestive heart failure, severe left ventricular systolic function, previous myocardial injury or acute coronary syndrome, cardiogenic shock and renal failure.

*MACE, major adverse cardiac events.*

**TABLE 6:** Cox regressions models—advanced time periods compared to the reference time period (2008–2010).

Outcome	HR (95% CI)	*P-*value	*P* for trend
1 year death univariate			0.778
2011–2013	1.06 (0.81–1.39)	0.662	
2014–2016	0.98 (0.75–1.28)	0.892	
2017–2019	0.99 (0.77–1.27)	0.933	
1-year death multivariate*			0.572
2011–2013	0.92 (0.70–1.21)	0.578	
2014–2016	0.98 (0.75–1.28)	0.901	
2017–2019	0.90 (0.70–1.16)	0.417	
1-year MACE univariate			< 0.001
2011–2013	1.05 (0.87–1.26)	0.609	
2014–2016	0.78 (0.64–0.95)	0.014	
2017–2019	0.77 (0.64–0.92)	0.005	
1-year MACE multivariate*			0.002
2011–2013	1.02 (0.85–1.24)	0.776	
2014–2016	0.83 (0.69–1.02)	0.073	
2017–2019	0.79 (0.66–0.96)	0.015	

*Adjusted to—age, hypertension, diabetes mellitus, congestive heart failure, severe left ventricular systolic function, previous myocardial injury or acute coronary syndrome, cardiogenic shock and renal failure.

*MACE, major adverse cardiac events.*

## Discussion

In the current study, from a large cohort of 20,301 consecutive patients, we observed an increased incidence of C-PCI procedures, with a trend toward reduced 1-yaer MACE rate following complex PCI. One-year mortality rates remained unchanged. To our knowledge, the current research represents the largest single center C-PCI registry, comparing trends over a 12-year period.

Our study demonstrates a gradual rise in the rate of C-PCI procedures over the last decade. This observation could be explained by the evolution of PCI over that time period, coupled with the rising age and increasing comorbidity profile of our patient population (11). Indeed, more patients are offered PCI in lieu of CABG owing to the progressively narrowing gap in treatment effect between PCI and CABG (4). On the other end, more people who would have been treated conservatively previously are now offered revascularization with PCI. Analyzing the data, the following patterns emerged—a gradual increase in LM and bifurcation interventions, and significant decrease in SVG and CTO intervention procedures. Several explanations apply: First, PCI for left main disease of low or intermediate anatomic complexity has been studied extensively in the past decade and was shown to have comparable outcomes with CABG (12, 13). Second, both scientific data and clinical expertise, which accumulated during the past decade, has dramatically increased the number of bifurcation procedures. The recent NORDIC-BALTIC (14) trial reinforced the findings of earlier BBC (15) and CACTUS (16) trials, favoring provisional side branch stenting, without the routine stenting of both main and side branches in true bifurcations.

We hypothesize that the simplification of bifurcation stenting has led to a wider application of PCI in bifurcation lesions, previously treated conservatively. Additionally, contemporary bifurcation stenting technique emphasize side-branch wire protection and preservation which may lead to a more “inclusive” definition of bifurcation lesion. While provisional stenting is favored in non-LM disease, some evidence supports the upfront 2-stent DK-crush technique in LM disease. The evolution of bifurcation stenting: classic crush—classic crush with kissing balloon inflation (KBI)—mini crush—DK crush—nano crush (17), could by itself lead to increased rates of C-PCI procedures as it offers new solution for complex coronary anatomy. In keeping with the findings of these trials, our research shows a gradual increase in the amount of bifurcation procedures over the last decade.

In contrast, CTO and SVG interventions have been steadily declining over that period of time. Despite the FREEDOM (18) trial which demonstrated CABG superiority over PCI for patients with multivessel CAD and DM, the total amount of PCIs is on the rise with a parallel decline in CABG (19). As described earlier, this phenomenon might be explained by the combination of worsening risk profile in patient eligible for revascularization combined with practical advances in C-PCIs in the form of cumulative experience, new techniques and more efficient devices. Moreover, the revolution of transcatheter aortic valve replacement (TAVR) over the last decade has diminished the role of CABG even further. CAD and aortic stenosis frequently coexist; hence PCI is frequently pursued pre-TAVR after discussions between the patient and the Heart Team (20). As to SVGs in particular, their use has been declining steadily over the past decade due to their worse outcomes as compared to arterial grafts (21, 22). Moreover, when comparing PCI of a diseased native artery with PCI of an SVG in patients with previous CABG who require PCI, SVG PCI has worse outcomes as shown by Redfors et al. (23), patient who underwent SVG PCI had higher rates of cardiac death, stent thrombosis, ischemia-driven target-vessel revascularization, and overall MACE at 2 years than did those who underwent PCI of the native vessel, whenever it is feasible. Furthermore, as was shown by Brilakis et al. (24), SVG PCI had higher rates of in hospital death, no-reflow, periprocedural MI, and cardiogenic shock as compared with PCI of the native vessel in patient with prior CABG. Hence, decrease in the number of CABGs, combined with the decreased use of SVGs during these surgeries, and the worse outcomes as compared to native vessel intervention, may explain the gradual decline in SVG intervention during our follow up.

The principal rational of CTO-PCI is to improve symptoms (25). It is defined as a total occlusion in a coronary artery with non-collateral Thrombolysis in Myocardial Infarction (TIMI) flow grade 0 of at least 3-month duration. CTO-PCI has evolved dramatically in both effectiveness and safety over the last decade and is now a standard complex procedure with a success rate over 90%, in highly experienced centers (26). Contrary to recently published data, our study shows a gradual decrease in CTO procedures. This decline may represent a more selective utilization of CTO-PCI in our institution owing to the absence of evidence supporting survival benefit (27). Lastly, is should be mentioned that *ad hoc* PCI of CTOs is not uncommon practice in our institution. Some lesions which could have been considered CTOs requiring planned prolonged procedures in the early time periods, may have been easily crossable with the more contemporary equipment and hence not classified as CTO.

## Limitations

This study has several limitations. First, although all data were collected prospectively, we used a single-center observational design which has the inherent limitations associated with a non-randomized comparison. Second, we decided to focus selectively on several important domains of C-PCI: LM, CTO, SVG or bifurcation intervention, and follow their temporal trends. Third, our data did not include either Medina classification for bifurcation lesions nor J-CTO scores for CTOs.

## Conclusion

Rates of C-PCIs are on the rise, with worse overall outcomes, including higher mortality, as compared to non-complex procedures. Although MACE and TVR decreased significantly throughout the years, acute MI and death remained unchanged. As the complexity of procedures increases, so does the need for a deeper understanding of its pathophysiology, and the need to synergize between complex invasive intervention and secondary prevention during follow up.

## Data Availability Statement

The raw data supporting the conclusions of this article will be made available by the authors, without undue reservation.

## Ethics Statement

The studies involving human participants were reviewed and approved by Ethics Committee of the Rabin Medical Center, in compliance with the Declaration of Helsinki. Written informed consent for participation was not required for this study in accordance with the national legislation and the institutional requirements.

## Author Contributions

MK wrote the first and final draft of the manuscript. SV, HV-A, and GG collected data and contributed to the analysis. TB organized the database. AS, PC, GW, and YT wrote sections of the manuscript. LP and RK contributed to the design of the study. AL conceived and designed the analysis and reviewed the manuscript. All authors contributed to manuscript revision, read, and approved the submitted version.

## Conflict of Interest

The authors declare that the research was conducted in the absence of any commercial or financial relationships that could be construed as a potential conflict of interest.

## Publisher’s Note

All claims expressed in this article are solely those of the authors and do not necessarily represent those of their affiliated organizations, or those of the publisher, the editors and the reviewers. Any product that may be evaluated in this article, or claim that may be made by its manufacturer, is not guaranteed or endorsed by the publisher.

## References

[1] Giustino G Chieffo A Palmerini T Valgimigli M Feres F Abizaid A Efficacy and safety of dual antiplatelet therapy after complex PCI. *J Am Coll Cardiol.* (2016) 68:1851–64. 10.1016/j.jacc.2016.07.760 27595509

[2] Généreux P Giustino G Redfors B Palmerini T Witzenbichler B Weisz G Impact of percutaneous coronary intervention extent, complexity and platelet reactivity on outcomes after drug-eluting stent implantation. *Int J Cardiol.* (2018) 268:61–7. 10.1016/j.ijcard.2018.03.103 30041804

[3] Baber U. Defining PCI complexity in the contemporary DES era: clarity or confusion? *Int J Cardiol.* (2018) 268:94–5. 10.1016/j.ijcard.2018.05.044 30041807

[4] Lee PH Ahn JM Chang M Baek S Yoon SH Kang SJ Left main coronary artery disease. *J Am Coll Cardiol.* (2016) 68:1233–46. 10.1016/j.jacc.2016.05.089 27609687

[5] Valle JA Tamez H Abbott JD Moussa ID Messenger JC Waldo SW Contemporary use and trends in unprotected left main coronary artery percutaneous coronary intervention in the united states: an analysis of the national cardiovascular data registry research to practice initiative. *JAMA Cardiol.* (2019) 4:100. 10.1001/jamacardio.2018.4376 30601910 PMC6439629

[6] Nagarajarao HS Ojha CP Mulukutla V Ibrahim A Mares AC Paul TK. Current use and trends in unprotected left main coronary artery percutaneous intervention. *Curr Cardiol Rep.* (2020) 22:16. 10.1007/s11886-020-1268-8 32036460

[7] Boukantar M Loyeau A Gallet R Bataille S Benamer H Caussin C Angiography and percutaneous coronary intervention for chronic total coronary occlusion in daily practice (from a large French registry [CARDIO-ARSIF]). *Am J Cardiol.* (2019) 124:688–95. 10.1016/j.amjcard.2019.05.062 31307663

[8] Gallone G D’Ascenzo F Conrotto F Costa F Capodanno D Muscoli S Accuracy of the PARIS score and PCI complexity to predict ischemic events in patients treated with very thin stents in unprotected left main or coronary bifurcations. *Catheter Cardiovasc Interv.* (2021) 97:28972. 10.1002/ccd.28972 32438488

[9] Endo A Kawamura A Miyata H Noma S Suzuki M Koyama T Angiographic lesion complexity score and in-hospital outcomes after percutaneous coronary intervention. *PLoS One.* (2015) 10:e0127217. 10.1371/journal.pone.0127217 26121583 PMC4487684

[10] Thygesen K Alpert JS Jaffe AS Chaitman BR Bax JJ Morrow DA Fourth universal definition of myocardial infarction (2018). *Eur Heart J.* (2019) 40:237–69. 10.1093/eurheartj/ehy462 30165617

[11] Vora AN Dai D Gurm H Amin AP Messenger JC Mahmud E Temporal trends in the risk profile of patients undergoing outpatient percutaneous coronary intervention: a report from the national cardiovascular data registry’s CathPCI registry. *Circ Cardiovasc Interv.* (2016) 9:3070. 10.1161/CIRCINTERVENTIONS.115.003070 26957417

[12] Stone GW Kappetein AP Sabik JF Pocock SJ Morice MC Puskas J Five-year outcomes after PCI or CABG for left main coronary disease. *N Engl J Med.* (2019) 381:1820–30. 10.1056/NEJMoa1909406 31562798

[13] Serruys PW Morice MC Kappetein AP Colombo A Holmes DR Mack MJ Percutaneous coronary intervention versus coronary-artery bypass grafting for severe coronary artery disease. *N Engl J Med.* (2009) 360:961–72. 10.1056/NEJMoa0804626 19228612

[14] Kumsars I Holm NR Niemelä M Erglis A Kervinen K Christiansen EH Randomised comparison of provisional side branch stenting versus a two-stent strategy for treatment of true coronary bifurcation lesions involving a large side branch: the Nordic-Baltic Bifurcation Study IV. *Open Heart.* (2020) 7:e000947. 10.1136/openhrt-2018-000947 32076558 PMC6999681

[15] Hildick-Smith D de Belder AJ Cooter N Curzen NP Clayton TC Oldroyd KG Randomized trial of simple versus complex drug-eluting stenting for bifurcation lesions: the british bifurcation coronary study: old, new, and evolving strategies. *Circulation.* (2010) 121:1235–43. 10.1161/CIRCULATIONAHA.109.888297 20194880

[16] Colombo A Bramucci E Saccà S Violini R Lettieri C Zanini R Randomized study of the crush technique versus provisional side-branch stenting in true coronary bifurcations: the CACTUS (coronary bifurcations: application of the crushing technique using sirolimus-eluting stents) study. *Circulation.* (2009) 119:71–8. 10.1161/CIRCULATIONAHA.108.808402 19103990

[17] Raphael CE O’Kane PD Johnson TW Prasad A Gulati R Sandoval Y Evolution of the crush technique for bifurcation stenting. *JACC.* (2021) 14:2315–26. 10.1016/j.jcin.2021.08.048 34736729

[18] Farkouh ME Domanski M Sleeper LA Siami FS Dangas G Mack M Strategies for multivessel revascularization in patients with diabetes. *N Engl J Med.* (2012) 367:2375–84. 10.1056/NEJMoa1211585 23121323

[19] Blumenfeld O Na’amnih W Shapira-Daniels A Lotan C Shohat T Shapira OM. Trends in coronary revascularization and ischemic heart disease–related mortality in Israel. *JAHA.* (2017) 6:e004734. 10.1161/JAHA.116.004734 28213569 PMC5523769

[20] Lahoud R Dauerman HL. Fall and rise of coronary intervention. *JAHA.* (2020) 9:16853. 10.1161/JAHA.120.016853 32458708 PMC7429013

[21] Carrel T Winkler B. Current trends in selection of conduits for coronary artery bypass grafting. *Gen Thorac Cardiovasc Surg.* (2017) 65:549–56. 28795296 10.1007/s11748-017-0807-8

[22] Kheifets M Vaknin-Assa H Greenberg G Assali A Kornowski R Perl L. Outcomes of primary percutaneous cardiac intervention for ST elevation myocardial infarction with a saphenous vein graft culprit. *Catheter Cardiovasc Interv.* (2020) 96:28662. 10.1002/ccd.28662 31868317

[23] Redfors B Généreux P Witzenbichler B McAndrew T Diamond J Huang X Percutaneous coronary intervention of saphenous vein graft. *Circ Cardiovasc Intervent.* (2017) 10:e004953. 10.1161/CIRCINTERVENTIONS.117.004953 28495896

[24] Brilakis ES O’Donnell CI Penny W Armstrong EJ Tsai T Maddox TM Percutaneous coronary intervention in native coronary arteries versus bypass grafts in patients with prior coronary artery bypass graft surgery. *JACC.* (2016) 9:884–93. 10.1016/j.jcin.2016.01.034 27085582

[25] Brilakis ES Mashayekhi K Tsuchikane E Abi Rafeh N Alaswad K Araya M Guiding principles for chronic total occlusion percutaneous coronary intervention: a global expert consensus document. *Circulation.* (2019) 140:420–33. 10.1161/CIRCULATIONAHA.119.039797 31356129

[26] Konstantinidis NV Werner GS Deftereos S Di Mario C Galassi AR Buettner JH Temporal trends in chronic total occlusion interventions in Europe: 17 626 procedures from the European registry of chronic total occlusion. *Circ Cardiovasc Interven.* (2018) 11:6229. 10.1161/CIRCINTERVENTIONS.117.006229 30354635

[27] Lee SW Lee PH Ahn JM Park DW Yun SC Han S Randomized trial evaluating percutaneous coronary intervention for the treatment of chronic total occlusion: the DECISION-CTO trial. *Circulation.* (2019) 139:1674–83. 10.1161/CIRCULATIONAHA.118.031313 30813758

